# Psychiatrists, Penguins and Pelicans: media psychiatrists and psychiatrists in the media

**DOI:** 10.1192/bjb.2025.10125

**Published:** 2026-04

**Authors:** Gavin Miller, Gordon David Lyle Bates

**Affiliations:** 1 Critical Studies, Glasgow University, Glasgow, UK; 2 Royal College of Psychiatrists, London, UK

**Keywords:** History of psychiatry, ethics, anthropology, clinical governance, education and training

## Abstract

To provide a useful contextual backdrop to an exhibition at the Royal College of Psychiatrists this summer, we used a question and answer format to summarise the thoughts of its curator, Gavin Miller. Gavin has chosen 12 books published by Penguin between 1949 and 1975 to illuminate the relationship between psychiatrists, psychologists, psychotherapists and the British media. He reflects on the opportunities and pitfalls that come with the association, the motivations of previous writers and provides practical advice for any media psychiatrists considering such a role in the future. The exhibition is open to visitors to the College building in London.

Over the summer of 2025, the Royal College of Psychiatrists will house a new exhibition on publicly prominent psychiatrists known primarily for their media work. Despite the recent surge of interest in mental health matters, particularly on social media platforms, there are few if any contemporary specialists with the kind of profile that was previously enjoyed by psychiatrists like R. D. Laing, Anthony Clare, William Sargant and David Stafford-Clark. Today, there is arguably more institutional control: the College has its own press office and actively recruits doctors to respond to media queries and provides guidelines and training for those who wish to offer their opinions to the general public (https://www.rcpsych.ac.uk/news-and-features/media-centre/public-education-handbook). Given that the more maverick pioneers of this work were writing and appearing on television and radio in the 1950s to the 1980s, it is quite possible that the majority of College members will not even have heard of most of them, so why should we be interested or visit the display?

This was among the questions that I put to Gavin Miller, Reader in Contemporary Literature and Medical Humanities at Glasgow University. It is Gavin who has researched, collated and curated the information for the exhibition. He has previously written for the *BJPsych Bulletin* on a related theme: ‘Beyond a literacy model for psychiatry in the mass media’.^1^

## Gordon Bates (Historian in Residence): What first attracted you to this topic?

**GM:** I became interested in R. D. Laing’s career as an author. He became a public figure in the 1960s and 1970s through his writing, and Glasgow University holds his papers in our archives – it’s one of our largest and most significant contemporary collections. The papers gave me a sense of Laing as a working author, as well as a radical psychiatrist and countercultural guru. He published several works with Penguin Books, including his countercultural classic, *The Politics of Experience*. I followed this thread to the Penguin Archive at Bristol University, where I’ve been able to find out more about Laing’s authorial career, as well as that of many other Penguin psychiatrists, psychotherapists and psychologists.

One of the most interesting figures in the Penguin Archive was David Stafford-Clark, who wrote a highly successful Pelican, *Psychiatry To-day* (1952), and *Psychiatry for Students* (1964), which remained in print until the 1970s and 1990 respectively. Pelican is the non-fiction imprint of Penguin books which publishes on academic topics for a broader audience. Stafford-Clark in the late 1950s and early 1960s was a prominent broadcaster and minor celebrity whose career is documented in the BBC Written Archives Centre. The more I found out about Stafford-Clark, the more I was struck by the analogy with Laing. Stafford-Clark was far more of an establishment figure, serving in the RAF and holding a consultant post at Guy’s hospital. He preceded Laing by about a decade, but the same career pattern is evident. Both were charismatic figures, adept in the media context of their time. Both used their psychiatric authority to carve out a wider career as a public commentator and celebrity. They both suffered a painful fall from celebrity – and they both wrote poorly received poetry.

It’s easy to offer a psychologised account of Laing’s public rise and fall in the media (e.g. an ‘addiction to fame’), particularly given his later personal difficulties, including alcohol addiction. But the comparison with Stafford-Clark shows that explanation to be incomplete. Both Laing and Stafford-Clark were pulled into a media and celebrity economy that needed them, but which increasingly offered diminishing returns as they became predictable features of the media landscape of television, radio and books. Audiences wearied of seeing the same faces again and again when psychiatric topics were on the agenda. Laing and Stafford-Clark were also somewhat unappealing to female audiences, who were often interested in expert perspectives from women, including Penguin authors such as the psychologist Penelope Leach and the lay psychoanalyst Juliet Mitchell.

## GB: Where else has the presentation been exhibited and what has been the response?

**GM:** I ran an earlier exhibition in autumn of 2022 at University of Stirling Library, which has a large collection of Penguin first editions, as well as a thriving context in publishing studies. That exhibition had a parallel online version as well. The exhibition audience were also surprised by the extent to which these titles, and their authors, addressed contentious social and political topics that might seem well beyond the realm of psychiatry or psychology, such as Marxist revolution, conflict in Northern Ireland or sexual mores. I also learned from audience feedback that they were most surprised by the extent to which authorship was collaborative to some degree. Many of these titles were extensively rewritten at Penguin’s request (or instruction) with a view to both style and content, the latter particularly where it strayed beyond the limits of psychiatry. Much of this work was done by female editors, some of whom might have been better credited as co-authors given the extent of the collaboration. One of these hidden figures (who so far remains nameless) incurred the wrath of psychiatrist J. A. C. Brown when she constructively criticised the quality of his historiography in drafts of *Techniques of Persuasion* (1963). In lengthy harangues to his commissioning editor, Brown denounced her as an underling who is ‘too clever by half’, ‘incompetent’ and no better than ‘a garage mechanic’. Some of these female editors can, though, be identified and retrospectively credited. The substantive contribution of Dorothy F. Paddon, a former Tavistock employee and freelance editor, runs through several Penguin titles, including Andrew Crowcroft’s *The Psychotic: Understanding Madness* (1967), Maxwell Jones’s *Social Psychiatry* (1968) and Anthony Ryle’s *Student Casualties* (1969). Paddon remains a shadowy figure, though, and if any readers know more about her, I would be very interested.

## GB: What is the format of the exhibition?

**GM:** There will be two long display cabinets containing a physical exhibition of 12 Penguin paperbacks, each with an interpretation. Elsewhere in the building, we will have a monitor display that has a digital version of the same exhibition: a carousel of slides showing an image of each book plus its interpretation. There will be plenty of opportunities to give feedback on the exhibition via a QR code, which will be displayed prominently, or by asking for a paper form. It’s important that I get feedback so I can evidence its ‘impact’. Like every UK academic, I need to accumulate evidence for the Research Excellence Framework – and for my own career development.

## GB: Are there any commonalities between the psychiatrists that you chose?

**GM:** They’re almost all men, unfortunately, which reflects the period that’s most accessible in the archival sources. It takes another generation before female psychiatrists and professional psychologists really start to feed through into the public sphere, and as new female editors at Penguin begin to look beyond the old boys’ club. The female authors of the 1950s to 1970s tended to be lay analysts, such as Juliet Mitchell and Frieda Fordham, with the child psychiatrist Sula Wolff being an honourable exception. Even Penelope Leach, author of the highly successful *Babyhood* (1974), was a psychologist rather than a psychiatrist. The sole female author in the exhibition, Rona Fields, was a clinical psychologist from the United States – but one who had very sharp disagreements with psychiatry in Northern Ireland during the height of the Troubles. (Fields had claimed that the inflammatory policy of interment launched in 1971 by the British Army was an intentional experiment upon the Catholic community, intended to foment factional violence – and, moreover, that local health professionals and psychological researchers were dupes who could be counted on for victim-blaming explanations in terms of cultural and social conflicts, aggressive needs and so forth.) A further difficulty in achieving greater gender balance is not just Penguin’s list but also the content of editorial files. They are immensely unpredictable in scope and content, and only a few generate compelling narratives.

## GB: Can the relationship between psychiatry, the media and the public ever be mutually beneficial?

**GM:** Undoubtedly. There is much everyday media work where broadcasters (or other intermediaries), the public and the psychiatric profession benefit. My feeling is that the best work is done when psychiatry is represented by a diversity of clinicians, so that intra-professional dissensus is also represented, and so that no single figure, or group of star performers, comes to stand for the profession. But the mass media has its own logic…

## GB: What do you think that the public and media expect from a psychiatric expert?

**GM:** To take the media first. They, of course, want someone who is a capable performer (doesn’t gabble, nor wave their hands in front of their face, and doesn’t get defensive or aggressive) and who has a suitable qualification to be giving an opinion in print or in broadcast or online media. But the media also think about representing diversity in the profession, the demographics of their audience and how they can square the two. A further desire may be that the psychiatrist becomes an all-purpose expert in their field. This may not sit easily with psychiatric specialisation, of course, but the convenience of a reliable performer is appealing.

The public want a psychiatrist who can maintain a parasocial relationship – at least if the psychiatrist is to be anything more than a one-off contributor. A radio or TV psychiatrist is a guest in the viewer’s home and has to be able to maintain a level of companiable, non-hierarchical conversation. The viewer, remember, has the luxury of choice: they can switch off or change channel. Newer media such as streaming bring the psychiatrist into even more intimate proximity: you may find yourself chatting to the listener on the morning commute, in bed with them or sitting next to them in the bathroom. Again, this can encourage the media to turn to the same reliable performers who can deliver that feeling of everyday conversation. There is, though, an eventual limiting factor: the public will also tire of over-exposure. Too much concentrated media presence of the same face will turn them off.

## GB: Were there any surprising stories that you discovered?

**GM:** I don’t want to give spoilers regarding the exhibition itself. Here’s a story, though, from another Penguin: the psychiatrist D. J. West’s *Homosexuality* (1960). This was published under the Pelican brand, which was intended as an authoritative library of modern knowledge. The title was a ‘takeover’ – a paperback edition of an earlier (in this case 1955) hardback from another publisher – and it was commissioned in response to the Wolfenden Report, which had recommended in 1957 the legalisation of homosexuality between adult males.

At the time the Pelican edition met popular demand across British society for expertise on this topic. The book reprinted within a year and went into a later revised edition in 1968 after the 1967 Sexual Offences Act legalised homosexuality between males over 21 (or did so, at least, in England and Wales). But by the 1970s the book was showing its age. The gay liberation movement was displeased by West’s apparent position – seemingly reiterated in contemporaneous lectures – that there were psychiatric issues intrinsic to homosexuality, rather than because of a still largely homophobic society and culture. The issue was brought home directly to Penguin when a recently published Finnish book misused – and possibly plagiarised – some of West’s text (in translation) to buttress a religiously motivated condemnation of homosexuality by the State Church of Finland. Penguin kept out of the possible legal dispute in another jurisdiction, but their editorial staff and external advisors recognised that West’s book was out of date. In 1975 Penguin dropped the title from their list, even though it was still selling well and commercially viable.

It’s uncertain, though, whether Penguin knew that West was a gay man. The editorial files suggest that they didn’t. But, as West’s 2012 memoir *Gay Life: Straight Work* makes clear, West had been enjoying, even in the early 1950s, the many opportunities offered by London’s gay subculture. *Homosexuality* was therefore a careful compromise with the professional and legal norms of the period. Whatever West’s feelings about his sexual orientation, the book’s representation of homosexuals as an afflicted but harmless minority was a prudent tactic: it repudiated legal and moral condemnation, while also avoiding the professional and legal dangers of too enthusiastic a defence. Those risks were real: David Stafford-Clark’s opposition to legal discrimination against homosexuals led to career-threatening gossip in the early 1960s that he was closeted (he wasn’t). From our perspective, it’s too easy to adopt a present-day liberal perspective and to criticise West for not being progressive enough, while not recognising the realities of a gay psychiatrist writing on homosexuality in the mid-1950s.

## GB: Why should we visit the exhibition at the College?

**GM:** The exhibition gives a longer historical view on the opportunities – and risks – of the public role. There’s a temptation to think of contemporary problems as peculiar to the here and now, particularly because of the rise of new technologies such as the internet, which give potentially enormous exposure with very little editorial gatekeeping. The problem of today seems to be how to deliver authoritative expert knowledge to a public who are being potentially misled by self-appointed experts communicating through social media. But, equally, the media psychiatrists of the past saw a similar problem: how to bring expert knowledge to a public whose ideas about mental illness were formed by stigmatising myths that circulated in the lay community, such as stereotypes of the dangerous ‘madman’.

Nor should we be innocent about the motives of past experts. Writing a book for the public that vilified opponents within psychiatry and psychology could be a quick route to discrediting and disempowering professional rivals: this applies as much to Hans Eysenck’s opposition to psychoanalysis as to R.D. Laing’s criticism of biological psychiatry.

Moreover, any psychiatrist that excels in a media role will be faced with the flattering demand for more of their services: reliable performers with ‘star quality’ are hard to find. If you have a lurking ambition to be a performer or a celebrity, then media work will have an extra appeal.

## GB: Do you think there is still commercial space for the kind of product Penguin and Pelican produced, and how should it be adapted to today?

Penguin, or Penguin Random House as they are now, still have a healthy list of psychological titles, even in the UK (the list is longer, I think, in the US, where the market is larger). It’s intriguing to see some of the titles from the 1960s and 1970s are still in print: R. D. Laing’s *The Divided Self* and *The Politics of Experience*, for instance, or Eric Berne’s *The Games People Play*. However, there are a couple of clear shifts, even at first glance. One is the diversification of authority. Psychiatrists are still in the mix, of course, with Bessel Van Der Kolk’s *The Body Keeps the Score* as a contemporary popular classic. But there’s now a lot more representation from psychologists, GPs, psychotherapists, presenters, journalists and celebrities. This diversification extends to the authority accrued in the ‘expertise from experience’ which may be invoked by authors, including those whose identity is primarily as a psychiatric service user, ‘survivor’ and so forth. Patrick J. Kennedy and Stephen Fried, for instance, present a range of stories – including caregivers’ – in *Profiles in Mental Health Courage* (2024), published in Penguin Random House’s Dutton imprint.

The other big obvious change is the enormous increase in self-help books, which the original Pelican imprint avoided, and in fact found harder to sell – at least according to some of the file material I’ve seen. Self-help books often draw on the author’s experiential expertise, alongside any academic or professional expertise, or use stories about how other people have been helped. They may also integrate with a wider monetisation through add-ons like subscription newsletters or videos. It’s interesting to wonder whether a UK psychiatrist can easily furnish that kind of commercial product. As well as ethical concerns about using patient case histories in mass-market publishing, I also wonder how realistic it would be to expect of psychiatrists the intimate self-disclosure that the self-help genre encourages. How freely can a professionally active psychiatrist write about their own mental and personal crises – will disclosure imperil their fitness to practise or have other, informal repercussions?

## GB: What would be your advice to any psychiatrist considering a media role?

**GM:** That entrepreneurial dimension is very significant. Media exposure offers further opportunities beyond merely the role of the psychiatric expert. We can think of celebrity – even of a minor degree – as a kind of capital that can be traded on for further opportunities. Perhaps it gets you another role in journalism or a deal for that novel or memoir you’ve been working on, or moves you into a political role heading up or advising a committee.

Now, from within, this will no doubt feel like due reward for years of training and accumulated expertise. But one thing I’ve learned from studying Penguins is how tempting it is for psychiatrists to speak on a wide variety of matters that are outside not just their professional expertise but even the remit of psychiatry itself. Now, you may well feel that this is entirely acceptable – to discuss matters of morality or religion. At some points you are wearing your professional hat, so to speak. At other times you have taken it off and put on another hat, the mitre of a secular archbishop perhaps.

But while that’s perhaps clear to you, the audience can’t necessarily be expected to know which metaphorical hat you are wearing or when. Moreover, this private distinction may be publicly obscured – precisely because the new role draws upon your medical authority. Could we really say, for instance, that Anthony Clare’s interviews for *In the Psychiatrist’s Chair* were psychiatric? They seem more like a psychiatric gloss upon the increasingly intimate personal interviews that became expected of celebrities in the 1960s and 1970s. Arguably, an untrained journalist could do them just as well – but a skilled psychiatric interviewer helped to make these interviews legitimate.

These concerns indicate why I think professional guidance on media work should go beyond ethics narrowly conceived, or tips ‘n’ tricks on how to come over well on camera. As the exhibition shows, there are a range of complex and ambiguous problems that media work raises for psychiatry. Rather than thinking of ‘media ethics’, psychiatrists might instead think of ‘responsible media work’, which includes but goes beyond ethical issues such as patient confidentiality or eschewing long-range diagnosis. This broader concept might include, for instance, reflection on one’s personal motivations or indeed those of psychiatry as a profession. Such reflection could mean articulating not just the publicly admissible motivations, such as a desire to help and educate, but also recognising and negotiating with the Jungian shadow-side: accruing power and money, gaining the admiration of others, pursuing rivalries within psychiatry or between psychiatry and other professions, and so on. One might also ask about the wider social and cultural implications of media work: issues such as exclusion from public representation (have you pushed out a service user?), the formation of parasocial relationships (do you have fans?), to the moulding of media work by the contemporary ‘attention economy’ (are you clickbait?). Finally, it’s perhaps tempting to think mainly of the principles of responsible media work, and thereby to conclude with a checkbox list of ‘pillars’ that hold up the roof. But, in my view, responsible media work needs the developed judgement that comes with thinking about concrete cases embedded in their complex and uncertain historical contexts (we might think of my discussion of West’s *Homosexuality*, above). This exhibition may be a step towards curating some informative cases.

## Books featured in the exhibition

Oswald Schwarz*, *The Psychology of Sex* (1949)

David Stafford-Clark, *Psychiatry To-day* (1952)

John Bowlby, *Child Care and the Growth of Love* (1953)

J. A. C. Brown, *The Techniques of Persuasion: From Propaganda to Brainwashing* (1963)

Anthony Storr, *Sexual Deviation* (1964)

Peter Hays, *New Horizons in Psychiatry* (1964)

G. M. Carstairs, *This Island Now* (1964)

John Hinton, *Dying* (1967)

Andrew Crowcroft, *The Psychotic: Understanding Madness* (1967)

R. D. Laing, *The Politics of Experience and The Bird of Paradise* (1967)

Rona Fields**, *Society on the Run: A Psychology of Northern Ireland* (1973)

Moses Laufer***, *Adolescent Disturbance and Breakdown* (1975)

*urologist and physician

**psychologist

***social worker and psychoanalyst
