# Psychiatrists, Penguins and Pelicans: media psychiatrists and psychiatrists in the media – CORRIGENDUM

**DOI:** 10.1192/bjb.2025.10147

**Published:** 2026-04

**Authors:** Gavin Miller, Gordon David Lyle Bates

**Keywords:** History of psychiatry, ethics, anthropology, clinical governance, education and training, corrigendum

Since the original publication of this paper, the authors have realised that there is a small typographical error in the text which reads:

“Fields had claimed that the inflammatory policy of interment launched in 1971 by the British Army was an intentional experiment”

Whereas this should say ‘internment’ and read:

“Fields had claimed that the inflammatory policy of internment launched in 1971 by the British Army was an intentional experiment”

The authors apologise for this error.
